# A dual-responsive RhB-doped MOF probe for simultaneous recognition of Cu^2+^ and Fe^3+^

**DOI:** 10.1038/s41598-024-62177-x

**Published:** 2024-05-23

**Authors:** Teng Zhang, Rui Cao, Jingying Li, Hanxiao Tang, Hang Su, Weisheng Feng, Zhijuan Zhang

**Affiliations:** 1 College of Pharmacy, Henan University of Chinese Medicine, Jinshui East Road 156, Zhengzhou, 450046 China; 2 College of Chinese Medical Sciences, Henan University of Chinese Medicine, Zhengzhou, 450046 China; 3 Collaborative Innovation Center of Research and Development on the Whole Industry Chain of Yu-Yao, Zhengzhou, 450046 Henan China; 4 Institute of Mass Spectrometer and Atmospheric Environment, Jinan University, Guangzhou, 510632 China

**Keywords:** Ratiometric fluorescence, Sensing, Cu^2+^, Fe^3+^, Metal–organic framework, Environmental sciences, Chemistry, Engineering, Materials science

## Abstract

Based on the dual response of RhB@UiO-67 (1:6) to Cu^2+^ and Fe^3+^, a proportional fluorescent probe with (*I*_*392*_*/I*_*581*_) as the output signal was developed to recognize Cu^2+^ and Fe^3+^. Developing highly sensitive and selective trace metal ions probes is crucial to human health and ecological sustainability. In this work, a series of ratio fluorescent probes (RhB@UiO-67) were successfully synthesized using a one-pot method to enable fluorescence sensing of Cu^2+^ and Fe^3+^ at low concentrations. The proportional fluorescent probe RhB@UiO-67 (1:6) exhibited simultaneous quenching of Cu^2+^ and Fe^3+^, which was found to be of interest. Furthermore, the limits of detection (LODs) for Cu^2+^ and Fe^3+^ were determined to be 2.76 μM and 0.76 μM, respectively, for RhB@UiO-67 (1:6). These values were significantly superior to those reported for previous sensors, indicating the probe’s effectiveness in detecting Cu^2+^ and Fe^3+^ in an ethanol medium. Additionally, RhB@UiO-67 (1:6) demonstrated exceptional immunity and reproducibility towards Cu^2+^ and Fe^3+^. The observed fluorescence quenching of Cu^2+^ and Fe^3+^ was primarily attributed to the mechanisms of fluorescence resonance energy transfer (FRET), photoinduced electron transfer (PET), and competitive absorption (CA). This work establishes a valuable foundation for the future study and utilization of Cu^2+^ and Fe^3+^ sensing technologies.

## Introduction

Metal elements are essential for biological growth and development, and have significant effects on life systems^1^. Iron, for example, is extensively involved in oxygen absorption and transportation, hemoglobin formation, gene regulation, and cholesterol-like synthesis. Similarly, copper is crucial for human health and plays a vital role in maintaining biological growth, metabolism, and homeostasis. Nevertheless, an excess or deficiency of Cu^2+^ or Fe^3+^ can disrupt the physiological system and result in various diseases, seriously affecting people's lives and health^2–4^. Therefore, it is imperative to develop a technological solution capable of promptly and precisely identifying Cu^2+^ and Fe^3+^.

To date, several conventional approaches have been used to analyze trace metal ions, involving inductively coupled plasma mass spectrometry (ICP-MS)^5^, high-performance liquid chromatography (HPLC)^6^, atomic absorption spectrometry (ABS)^7^, and electrochemical analysis^8^. However, their practical implementation is constrained by their cumbersome operation and expensive equipment. Hence, an imperative requirement arises for a reaction-based technique that is both cost-effective and expeditious in detecting minute quantities of metal ions. Luminescent metal–organic frameworks (LMOFs) have recently garnered considerable interest owing to their numerous advantages, encompassing diverse luminous modes, rapid response, substantial specific surface area, and satisfactory modifiability^9^. This has resulted in extensive application of LMOFs throughout many fields, including sensing^10^, gas separation^11^, catalysis^12^, and drug transportation^13^. Particularly noteworthy is the remarkable potential in fluorescence sensing, encompassing the detection of metal ions^14^, small molecules^15^, gases^16^, volatile organic compounds (VOCs)^17^, and explosives^18^. For instance, Zhang et al. have effectively prepared a Tb-based MOF (Tb-TATB) to detect Cu^2+^ and Fe^3+^ ions, achieving limits of detection (LODs) of 2.92 μM and 4.84 μM, respectively^19^. In our previous work, an imidazole-based material (Ag/Zn-ZIF-8 (1:1)) was synthesized for the detection of Cu^2+^ and Fe^3+^, and the LODs reached 6.7 μM and 3.9 μM, respectively^20^. However, the efficacy of these fluorescent probes in detecting Cu^2+^ and Fe^3+^ was found to be unsatisfactory due to their reliance on the change in single-emission fluorescence intensity for sensing, which was often influenced by uncontrollable variables like photobleaching, light scattering, and concentration inhomogeneity^21^. Ratiometric fluorescent probes could effectively overcome these drawbacks due to their multi-occurrence self-calibration signals. Considering the excellent resultant stability and unique optical properties of Zr-MOF, combining Zr-MOF with fluorescent dyes is regarded as an intuitive and effective method, which not only restricts the migration and aggregation of fluorescent dye molecules by Zr-MOF but also generates peculiar fluorescent properties through the subject-object interaction^22^.

Based on the above factors, a series of ratiometric fluorescent probes were successfully prepared in this work by encapsulating rhodamine B (RhB) into UiO-67. In addition, Cu^2+^ and Fe^3+^, as hard acids, can easily coordinate with hard bases such as carboxyl groups^23,24^. The organic ligands biphenyl-4,4′-dicarboxylate (H_2_BPDC) and RhB have abundant carboxyl groups, which can easily coordinate with each other and Cu^2+^ and Fe^3+^, significantly improving the selectivity and detection limits of RhB@UiO-67 for Cu^2+^ and Fe^3+^^25^. The LODs of Cu^2+^ and Fe^3+^ for RhB@UiO-67 were 2.76 μM and 0.76 μM, respectively. Additionally, the probe was also shown to have excellent potential for sensing Cu^2+^ and Fe^3+^ in terms of its sensitivity, selectivity, immunity to interference as well as reproducible performance. Furthermore, possible sensing mechanisms have been systematically investigated. Consequently, this current study provides a straightforward and effective strategy for exploring the efficient sensing of trace metal ions (Cu^2+^ and Fe^3+^).

## Experimental

### Preparation of LMOFs

UiO-67 was prepared with adjustments based on previous literature^26^. ZrCl_4_ (0.5 mmoL, 116.52 mg), and H_2_BPDC (0.5 mmoL, 121.11 mg) were dissolved in 30 mL of DMF and subjected to sonication for 30 min to dissolve. Then, the resulting solution was transferred to a Teflon jar and heated at 120 ℃ for 24 h. The strip samples were subsequently cooled to room temperature and washed three times each with DMF and methanol. The white powder obtained was dried at 120 °C for 48 h, followed by further characterizations. Within this experimental procedure, a series of RhB@UiO-67 were synthesized with varying RhB/H_2_BPDC ratios of 1:2, 1:4, 1:6, 1:8, and 1:10, respectively. Additionally, the pore structure characteristics of these materials were assessed to ensure maximum surface area and pore volume.

### Fluorescence sensing experiments

The synthesized fluorescent probes were activated under vacuum at 120 °C for 12 h for fluorescence sensing. The fluorescence properties of UiO-67 and RhB@UiO-67 (1:6) in different solvents were investigated using a spectrophotometer (Hitachi F-7100) with excitation/emission slits of 5.0 nm and photomultiplier voltage of 300 V. The concentration of MOFs was 0.33 mg/mL in selectivity, titration, and immunity experiments.

### Density functional theory (DFT) calculation

In this experiment, geometry optimization, the energy levels of lowest unoccupied molecular orbitals (LUMOs) and highest occupied molecular orbitals (HOMOs) were evaluated using density functional theory (DFT) and optimized using Dmol3 of the Materials Studio 2019 package. The all-electron interaction theory (AER) potential accounts for the interaction between electrons and ions. All atoms were allowed to spin unrestricted during the structure optimization process. The GGA-PBE functional and DNP4.4 basis sets were used for the calculations. In the current study, the convergence criterion of the electronic self-consistent field (SCF) loop was set to be 10^–6^ with energy 10^–5^ Ha, force constant 0.002 Ha/Å, displacement 0.005 Å, and value of smearing 0.05 Ha^27,28^. Considering that the metal ions might interact with the carboxyl groups in the organic ligands, H_2_BPDC and RhB, to simplify the computational process, the optimized structure considered only a single carboxyl group and a metal ion on the ligand. All calculations were performed using a solvent model (ethanol) to obtain more accurate and reliable results.

### Analysis of real samples

First of all, 100 mg of *Alisma plantago-aquatica* L. was soaked in 50 mL of ethanol for 24 h and then filtered using a 0.22 μm filter membrane. Eventually, the samples were analyzed with the addition of different concentrations of Cu^2+^ and Fe^3+^ solutions (1 μM, 5 μM, and 10 μM) for three times.

## Results and discussion

### Characterization of RhB@UiO-67

**Figure 1:**
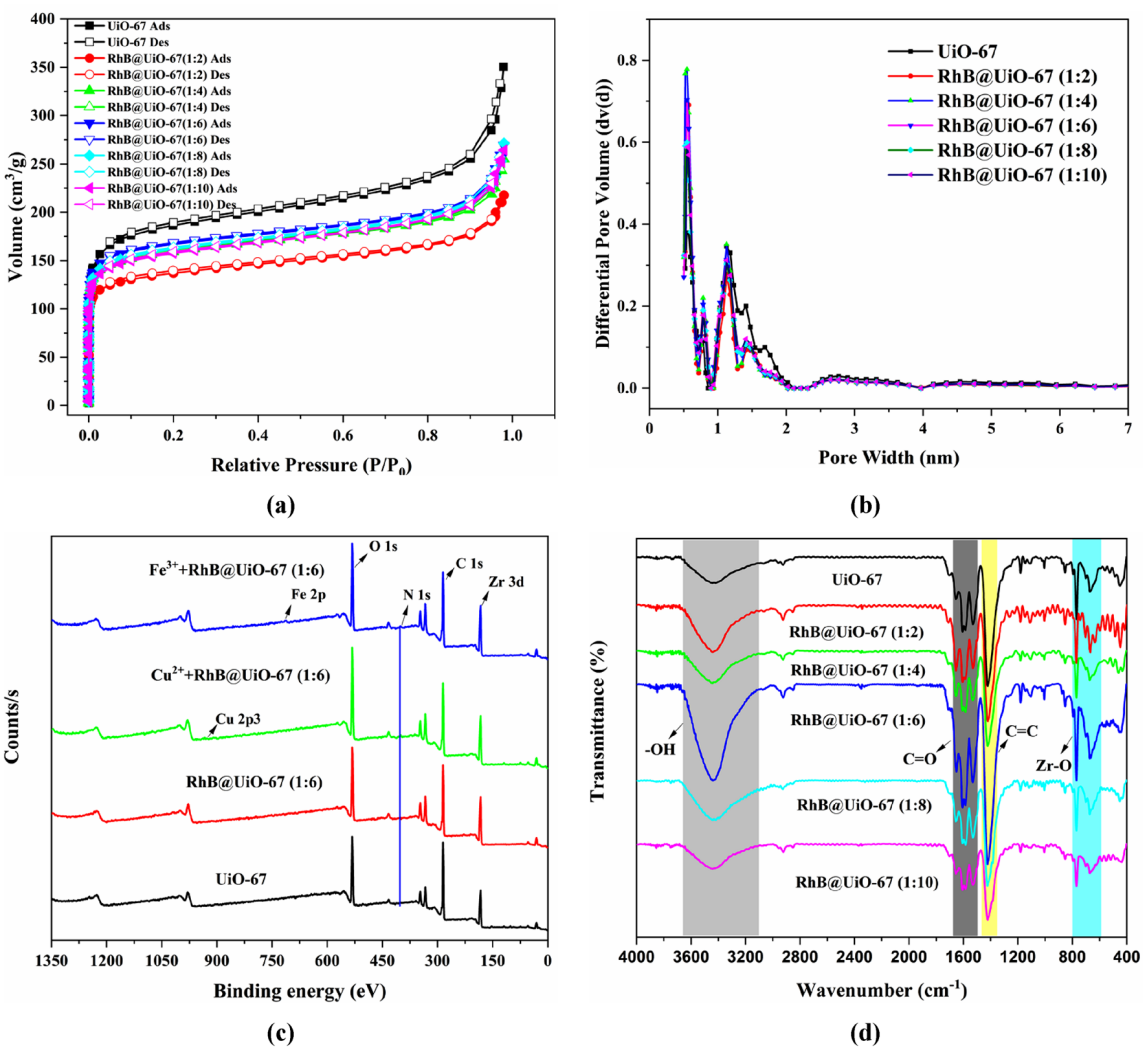
(**a**) N_2_ adsorption–desorption isotherms at 77 K. (**b**) NLDFT pore size distributions. (**c**) XPS spectra. (**d**) FTIR spectra between 4000 and 400 cm^−1^ of RhB@UiO-67 materials.

The N_2_ adsorption–desorption isotherms of obtained RhB@UiO-67 materials at 77 K were presented in Fig. 1a. Obviously, the adsorption capacity of N_2_ exhibits a rapid increase at lower relative pressures (p/p_0_), conforming to a typical type I isotherm, indicating the presence of numerous micropores in RhB@UiO-67^29^. Additionally, the NLDFT pore size distributions of RhB@UiO-67 materials were more uniform compared with those of UiO-67. And, in RhB@UiO-67, the BET surface areas were relatively small, possibly because RhB plugged the pore channels of UiO-67 (Fig. 1b). Meanwhile, the BET surface area increased followed by a decrease with an increase of RhB, reaching a maximum at RhB/H_2_BPDC of 1:6 (Table 1). This increase may be caused by the presence of new pore structure shapes, and the decrease may be caused by the continuous addition of RhB to disrupt the symmetry of the original UiO-67 leading to partial structural collapse and blockage of the pore channels^22,23^. RhB@UiO-67 (1:6) was chosen as a potential probe material for metal ion detection due to its significant specific surface area and pore volume.

**Table 1:** Parameters of porous structures for RhB@UiO-67 materials.

MOFs	BET (m^2^/g)	Langmuir (m^2^/g)	Micropore volume (cm^3^/g)	Total pore volume (cm^3^/g)	Average pore diameter (nm)
UiO-67	704	859	0.231	0.5423	3.078
RhB@UiO-67 (1:2)	522	759	0.175	0.3368	2.580
RhB@UiO-67 (1:4)	610	715	0.206	0.3946	2.584
RhB@UiO-67 (1:6)	641	749	0.219	0.4170	2.599
RhB@UiO-67 (1:8)	618	739	0.211	0.4198	2.716
RhB@UiO-67 (1:10)	597	722	0.202	0.4088	2.734

Several approaches were used to characterize the RhB@UiO-67. As shown in Fig. S1, SEM–EDS mapping of RhB@UiO-67 revealed that it had a uniform particle size and nanoscale structure, which facilitated a homogeneous dispersion in subsequent sensing experiments. In addition, compared with UiO-67, RhB@UiO-67 (1:6) exhibited a unique element N from RhB (Fig. S1), which indicated that RhB@UiO-67 (1:6) was successfully prepared. Interestingly, the XPS spectrum of RhB@UiO-67 (1:6) also demonstrated a peak at N 1s that was not present in UiO-67, which also proved that RhB@UiO-67 was successfully prepared (Fig. 1c)^30^.

The PXRD patterns of RhB@UiO-67 materials were very similar to that of pure UiO-67, indicating the well-maintained structure of UiO-67 after the introduction of RhB (Fig. S2). Therefore, it was likely that RhB was encapsulated within the voids rather than physically adsorbed on the surface of UiO-67. In addition, Fig. 1d demonstrated that the FTIR spectra of UiO-67 and RhB@UiO-67 were highly similar, confirming the encapsulation of RhB and the negligible effect of host–guest encapsulation on the structural integrity of UiO-67^31^. Furthermore, its exceptional thermal stability was demonstrated by its unchanged state at temperatures up to 450 °C (Fig. S3).

### Photoluminescence properties

#### Fluorescence performance of RhB@UiO-67

The solid-state fluorescence properties of the organic ligand (H_2_BPDC), vacuum-activated UiO-67, and RhB@UiO-67 were examined. The presence of abundant aromatic rings in the π-π conjugated system of the organic ligand resulted in a strong emission at 391 nm upon excitation at 322 nm but the fluorescence intensities of both UiO-67 and RhB@UiO-67 were weaker than that of H_2_BPDC due to the aggregation-induced quenching (Fig. S4a). A new fluorescence peak appeared at 582 nm when RhB was introduced. In addition, the fluorescence intensity of RhB@UiO-67 ligand out showed an initial enhancement followed by a weakening trend after the addition of RhB (Fig. S4b). The initial enhancement could be caused by the interaction between RhB and UiO-67. In contrast, the subsequent decrease resulted from the fluorescence quenching of RhB due to aggregation induction.

Additionally, the fluorescence behavior of RhB@UiO-67 (1:6) in various solvents (water, MeOH, EtOH, and DMF) was investigated at room temperature. It was evident that RhB@UiO-67 (1:6) demonstrated the best fluorescence behavior in ethanol (Fig S5a). Furthermore, the investigation of the fluorescence stability of RhB@UiO-67 (1:6) involved an examination of its relative fluorescence intensity at different time intervals and pH levels. The results demonstrated that the relative fluorescence intensity (*I*_*392*_*/I*_*581*_) of RhB@UiO-67 (1:6) remained consistent even after being immersed in ethanol for a duration of one week (Fig. S6c). Additionally, the relative fluorescence intensity of RhB@UiO-67 (1:6) showed minimal variation within the pH range of 3 to 10 (Fig. S6b). Afterward, the *I*_*392*_*/I*_*581*_ ratio of RhB@UiO-67 (1:6) was evaluated at different concentrations. Notably, the *I*_*392*_*/I*_*581*_ ratio initially increased with the concentration of RhB@UiO-67 (1:6), reaching its peak at 0.33 mg/mL (Fig. S5), before subsequently declining. Moreover, it was observed that the *I*_*392*_*/I*_*581*_ ratio remained essentially stationary at 60 s (Fig. S6a). Thus, the optimal concentration and incubation time for RhB@UiO-67 (1:6) were 0.33 mg/mL and 60 s, respectively.

#### Fluorescence detection of Cu^2+^ and Fe^3+^

**Figure 2:**
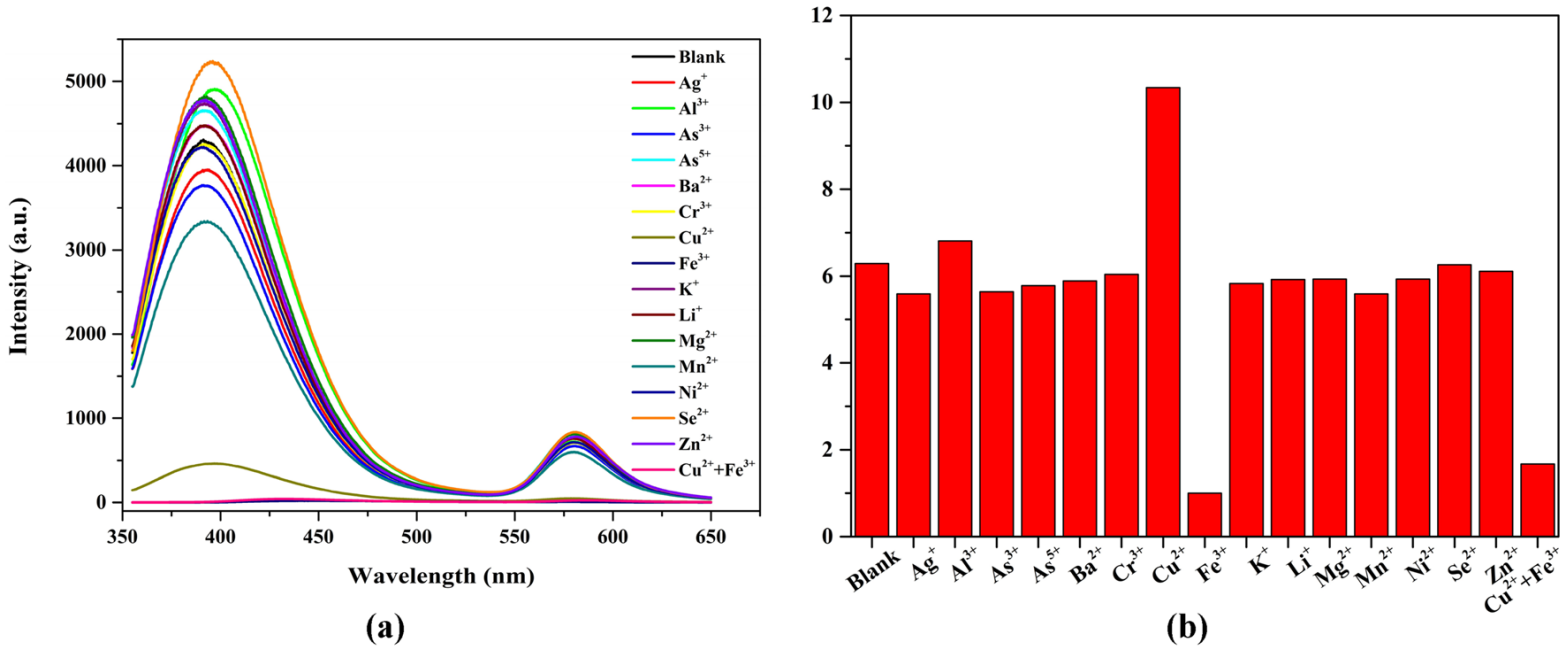
(**a**) Fluorescence responses of RhB@UiO-67 (1:6) (λex = 335 nm) (0.33 mg/mL) in the presence of various metal ions. (**b**) Relative fluorescence intensity of RhB@UiO-67 (1:6) toward various metal ions in ethanol solution.

To investigate the impact of RhB doping on the fluorescence properties of UiO-67, photoluminescence sensing experiments were conducted on a range of metal ions. Figure 2a illustrates that RhB@UiO-67 (1:6) presents remarkable selectivity towards Cu^2+^ and Fe^3+^ in comparison to other metal ions. The relative fluorescence intensity (*I*_*ligand*_*/I*_*RhB*_) of RhB@UiO-67 (1:6) exhibited a 64.4% increase for Cu^2+^ and an 84.1% decrease for Fe^3+^. However, when examining other potential interfering metal ions, the relative fluorescence intensity (*I*_*ligand*_*/I*_*RhB*_) of RhB@UiO-67 (1:6) did not change significantly compared to the blank solution (Fig. 2). Additionally, the fluorescence intensity of RhB@UiO-67 (1:6) experienced a decrease across all ligand wavelengths during the detection of selected metal ions (Cu^2+^ and Fe^3+^) in comparison to UiO-67 (Figs. 2a and S7a). Nevertheless, RhB@UiO-67 (1:6) exhibited a noteworthy increase in relative fluorescence intensity (*I*_*ligand*_*/I*_*RhB*_) compared to the blank solution when detecting Cu^2+^, which may be attributed to the interaction between Cu^2+^ and H_2_BPDC/ RhB. In addition, it is evident that the fluorescence intensity of RhB@UiO-67(1:6) for simultaneous Cu^2+^/Fe^3+^ sensing is lower than that for Cu^2+^ sensing, yet higher than that for Fe^3+^ sensing (Fig. S7a). This discrepancy may be attributed to the competition for energy transfer from RhB@UiO-67(1:6) to Cu^2+^ or Fe^3+^ in the context of simultaneous Cu^2+^/Fe^3+^ sensing.

**Figure 3:**
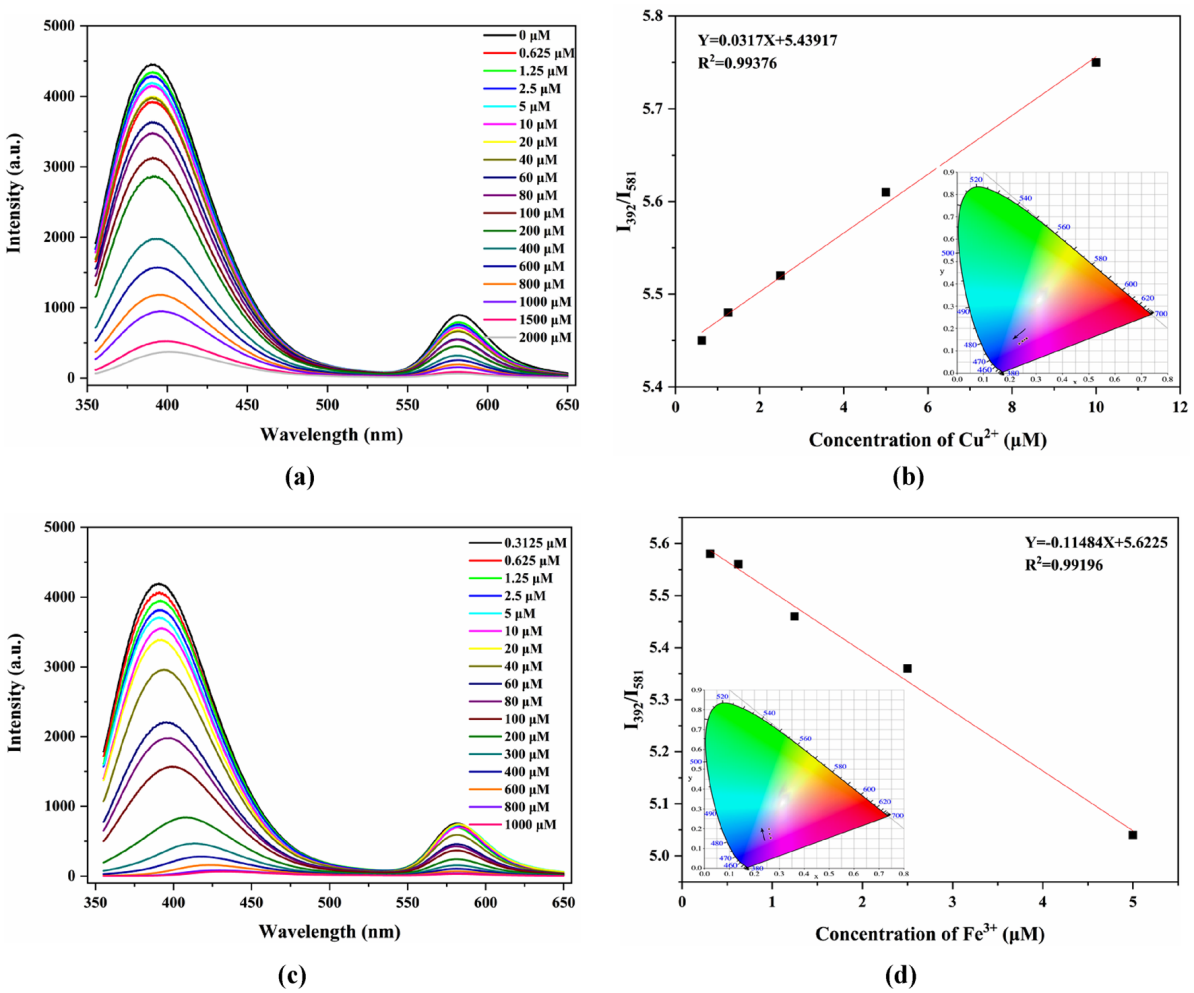
Fluorescence spectra and linear plot of RhB@UiO-67 (1:6) (λex = 335 nm) in the presence of various concentrations of Cu^2+^/Fe^3+^ in ethanol solution (**a**) and (**b**), Cu^2+^; (**c**) and (**d**), Fe^3+^.

The assessment of sensitivity plays a crucial role in the practical application of probes. To evaluate the sensitivity of RhB@UiO-67 (1:6) towards Cu^2+^ and Fe^3+^, fluorescence titration experiments were performed. The results indicated that RhB@UiO-67 (1:6) only displayed fluorescence behavior at 392 nm when detecting Cu^2+^ and Fe^3+^. Upon the introduction of RhB, RhB@UiO-67 (1:6) exhibited a new peak at 581 nm. Moreover, the fluorescence intensity of UiO-67 and RhB@UiO-67 (1:6) at 392 nm decreased progressively with the increase of Cu^2+^. Additionally, the fluorescence intensity of RhB@UiO-67 (1:6) at 581 nm also showed a similar trend, indicating an interaction between Cu^2+^ and the organic ligands H_2_BPDC and RhB (Figs. 3a and S8a). These observed fluorescence behaviors provided a valuable reference for internal calibration, effectively reducing interference in Cu^2+^ detection. Therefore, the ratio *I*_*392*_*/I*_*581*_ was deemed a self-calibrated signal ratio for RhB@UiO-67 (1:6). The Cu^2+^ concentration showed a strong linear correlation with the relative fluorescence value (*I*_*392*_*/I*_*581*_), as represented by the equation *I*_*392*_*/I*_*581*_ = 0.0317X + 5.43917 (X = 0–10 μM), with a high correlation coefficient (R^2^) of 0.99376. The LOD of Cu^2+^ by this probe was determined to be 2.76 μM (Fig. 3b), which was significantly lower than that of UiO-67 (17.63 μM) (Fig. S8b). In addition, the chromaticity coordinates (CIE coordinates) of RhB@UiO-67 (1:6) were determined based on the fluorescence spectra, revealing a color transition from pink to light purple with increasing Cu^2+^ concentration (inset of Fig. 3b).

The titration experiments conducted on RhB@UiO-67 (1:6) with Fe^3+^ demonstrated a concentration-dependent relationship in the emission spectra following excitation at 335 nm. The fluorescence intensity of RhB@UiO-67 (1:6) gradually decreased with increasing Fe^3+^ concentration (Fig. 3c), and the relative fluorescence intensity (*I*_*392*_*/I*_*581*_) of RhB@UiO-67 (1:6) strongly correlated with concentration. Notably, a significant linear correlation was observed when plotting the working curve of relative fluorescence values (*I*_*392*_*/I*_*581*_) against Fe^3+^ concentration: *I*_*392*_*/I*_*581*_ = − 0.11484X + 5.6225 (X = 0–5 μM), with a correlation coefficient (R^2^) of 0.99196. The LOD for RhB@UiO-67 toward Fe^3+^ was estimated to be 0.76 μM (Fig. 3d), which was significantly lower than that of the UiO-67 (15.62 μM) (Fig. S8d). Furthermore, the CIE coordinates of RhB@UiO-67 (1:6) revealed a transition in color from deep red to light red when the concentration of Fe^3+^ increased (inset of Fig. 3d).

In view of the above, it can be concluded that RhB@UiO-67 (1:6) possesses the potential to effectively monitor the Cu^2+^ and Fe^3+^ aspects through the utilization of a multi-response sensing model. Interestingly, RhB@UiO-67 (1:6) exhibited superior sensitivity compared to other probes designed to detect Cu^2+^ and Fe^3+^ (Table S1).

**Figure 4:**
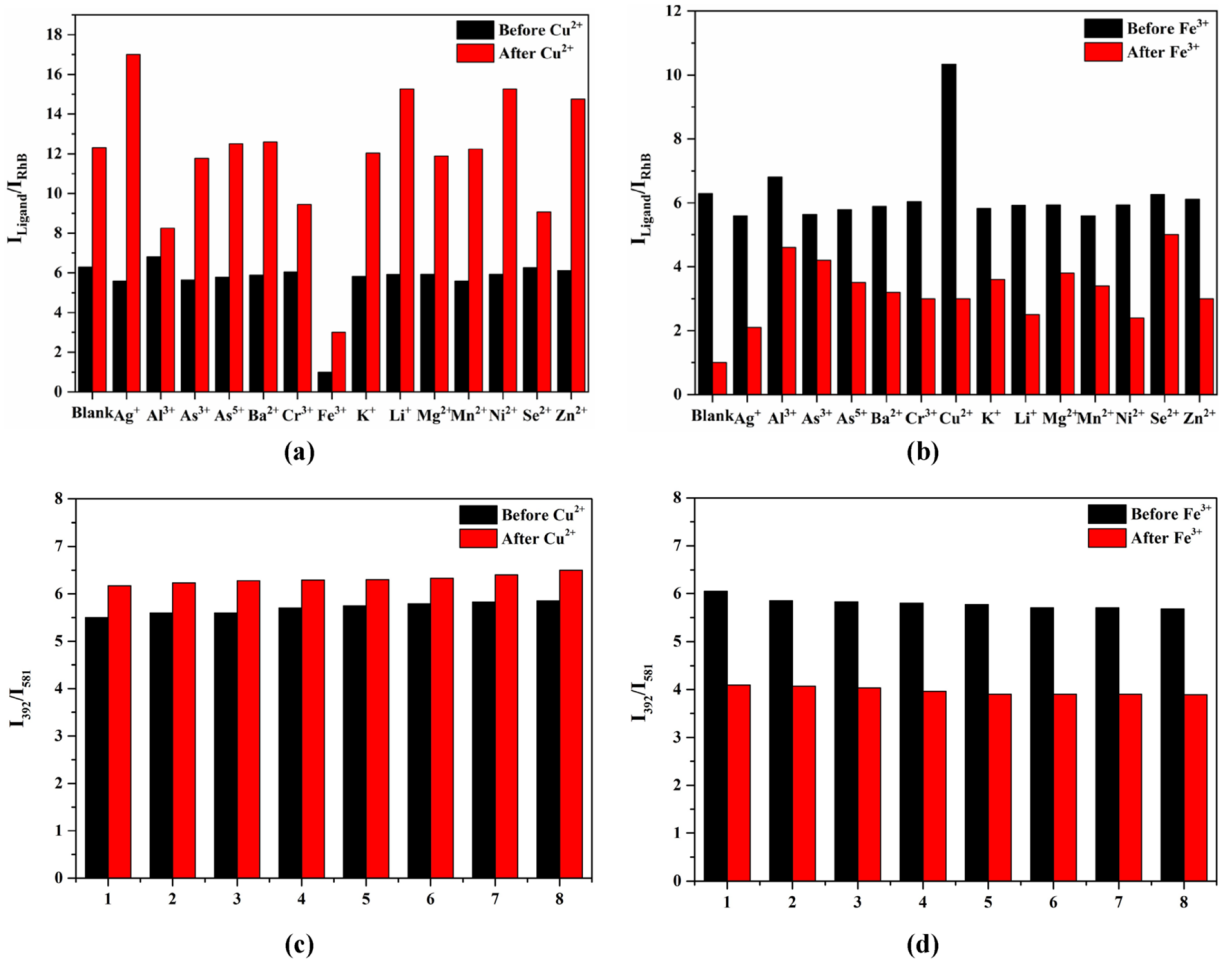
Relative fluorescence intensity of RhB@UiO-67 (1:6) upon the addition of (**a**) Cu^2+^ and (**b**) Fe^3+^ (10^–4^ mol/L) when various cations are present (10^–4^ mol/L) in ethanol; Regeneration properties of RhB@UiO-67 (1:6) after 8 cycles for (**c**) Cu^2+^and (**d**) Fe^3+^.

The probe must possess the capacity to detect the target analyte when other interfering ions are present. Consequently, anti-interference experiments were performed on RhB@UiO-67 (1:6). As shown in Fig. 4a and b, it is evident that the addition of Cu^2+^ and Fe^3+^ resulted in the quenching of RhB@UiO-67 (1:6) when coexisting with other metal ions. This finding suggests that RhB@UiO-67 (1:6) is less affected by other interfering ions when sensing Cu^2+^ and Fe^3+^. As a result, RhB@UiO-67 (1:6) shows promise for selectively sensing Cu^2+^ and Fe^3+^ in practical applications.

The ability to regenerate the probe material is also crucial in evaluating its actual sensing performance. To assess the regeneration capability, the probe material was washed with ethanol, followed by centrifugation and drying before conducting subsequent sensing experiments. The results presented in Fig. 4c and d demonstrate that the relative fluorescence intensity of RhB@UiO-67 (1:6) returned to its initial value after several consecutive sensing experiments. Furthermore, the pore structure of RhB@UiO-67 (1:6) remained essentially unchanged following 5 cycles of regeneration in Cu^2+^ and Fe^3+^ ion solutions, as depicted in Fig. S9a,b and Table S2. However, the BET-specific surface area of RhB@UiO-67 (1:6) began to decrease after 8 cycles, potentially due to the degradation of pore channels by Cu^2+^ or Fe^3+^ ions after 8 cycles, as shown in Fig. S9c,d and Table S2. These results indicate that the potential reusability of RhB@UiO-67 (1:6) in detecting Cu^2+^ and Fe^3+^ ions.

**Figure 5:**
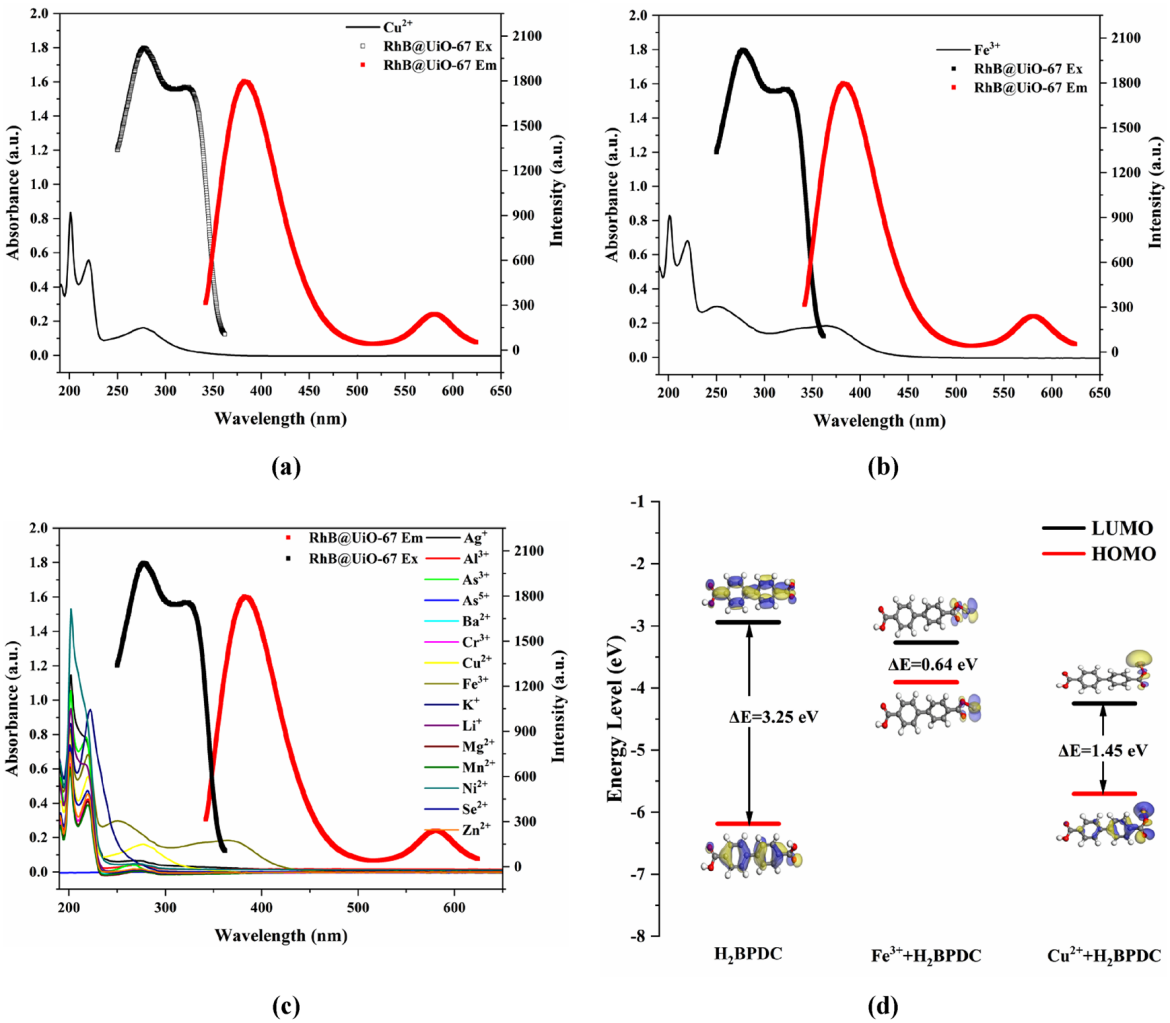
The UV–vis absorbance spectrum of Cu^2+^/Fe^3+^ (10^–4^ mol/L) and the photoluminescence spectra of RhB@UiO-67 (1:6). (**a**) Cu^2+^, (**b**) Fe^3+^. (**c**) UV–vis absorbance spectra of different cations in ethanol solution (10^–4^ mol/L) and the photoluminescence spectra of RhB@UiO-67 (1:6). (**d**) Frontal molecular orbital distributions of H_2_BPDC, Cu^2+^-H_2_BPDC, and Fe^3+^-H_2_BPDC calculated by DMol3.

To further understand the fluorescence quenching mechanisms toward Cu^2+^ and Fe^3+^, several experiments were conducted. First of all, Fig. 5 depicts the UV absorption profiles of the metal ions under investigation and the fluorescence curves of RhB@UiO-67 (1:6). It was found that among the metal ions under investigation, only the UV absorption profiles of Cu^2+^ and Fe^3+^ exhibited overlaps with the fluorescence excitation spectrum of RhB@UiO-67 (1:6), signifying the presence of competitive absorption (CA) between RhB@UiO-67 (1:6) and Cu^2+^/Fe^3+^^32^. Furthermore, the UV absorption profiles of Cu^2+^ and Fe^3+^ partially coincided with the fluorescence emission spectrum of RhB@UiO-67 (1:6), suggesting the occurrence of energy transfer between RhB@UiO-67 (1:6) and Cu^2+^/Fe^3+^. Moreover, the titration detection of Cu^2+^/Fe^3+^ experiments indicated that RhB@UiO-67 (1:6) functioned as a turn-off ratiometric fluorescent probe for both ions. This was evidenced by the observed energy transfer from RhB@UiO-67 (1:6) to Cu^2+^/Fe^3+^^33^. Additionally, the fluorescence lifetimes of RhB@UiO-67 (1:6) at different wavelengths were altered upon the introduction of different concentrations of Cu^2+^ and Fe^3+^ ions, demonstrating quenching mechanisms between RhB@UiO-67 (1:6) and Cu^2+^/Fe^3+^. The energy transfer efficiencies were determined through calculations based on Fig. S12 (Tables S3, S4)^34^. Thus, there was fluorescence resonance energy transfer (FRET) in the energy response between RhB@UiO-67 (1:6) and Cu^2+^/Fe^3+^.

Subsequently, FTIR analysis revealed the presence of a peak at 1650 cm^–1^, which can be attributed to the carboxylate stretching vibration. There was a blue shift of the peak at 1699 cm^–1^ owing to the interaction between Cu^2+^/Fe^3+^ ions and the carboxyl groups present in the organic ligand H_2_BPDC and RhB (Fig. S10)^35,36^. Furthermore, the XPS spectra revealed the presence of additional peaks, namely the Cu 2p peak at 935.55 eV, when RhB@UiO-67 (1:6) interacted with Cu^2+^ ions, and the Fe 2p peak at 722.13 eV, when RhB@UiO-67 (1:6) interacted with Fe^3+^ ions, which were absent in the RhB@UiO-67 (1:6) material (Fig. S11). Moreover, the high-resolution XPS spectrum of the O 1 s orbital exhibited a minimal change in the binding energy of the -OH bond and a minor variation in the metal-O (metal as Cu/Fe) bond after the interaction with Cu^2+^ and Fe^3+^. This observation suggests that Cu^2+^/Fe^3+^ ions interact with the carboxyl groups in the organic ligands H_2_BPDC and RhB^37^.

To further substantiate this proposed mechanism, the energies of LUMOs and HOMOs were calculated. The LUMOs of both Cu^2+^-H_2_BPDC and Fe^3+^-H_2_BPDC were found to be lower than that of the ligand, suggesting the occurrence of photoelectron transfer (PET) between RhB@UiO-67 (1:6) and Cu^2+^/Fe^3+^ (Fig. 5d)^38^.

#### Real samples detection

The reliability and adaptability of the method were further evaluated by the determination of Cu^2+^ and Fe^3+^ in *Alisma plantago-aquatica* L (one Chinese herbal medicine). The standard addition method was employed, and the results, presented in Table 2, exhibited recoveries ranging from 100.8 to 107.8% with the RSD below 2.73% (n = 3). These findings indicate that the method in the study serves as a robust platform for the detection of Cu^2+^ and Fe^3+^.

**Table 2:** Fluorescence detection of Cu^2+^ and Fe^3+^ in *Alisma plantago-aquatica* L (n = 3).

Metal ions	Add (μM)	Found (μM)	Recovery (%)	RSD (%)
Cu^2+^	1	1.07 ± 0.21	107.0 ± 21	1.01
	5	5.04 ± 0.01	100.8 ± 0.2	0.82
	10	10.09 ± 0.41	100.9 ± 4.1	1.15
Fe^3+^	1	1.06 ± 0.02	106.0 ± 2.0	1.29
	2.5	2.52 ± 0.2	100.8 ± 8.8	2.73
	5	4.96 ± 0.43	107.8 ± 8.6	1.62

## Conclusion

The ratiometric fluorescent probe RhB@UiO-67 prepared by the one-pot method was used for sensing trace amounts of Cu^2+^ and Fe^3+^ due to its unique fluorescence properties. Interestingly, RhB@UiO-67 (1:6) exhibited fluorescence behavior with fluorescence quenching for both Cu^2+^ and Fe^3+^. Furthermore, the RhB@UiO-67 (1:6) composite demonstrated exceptional immunity, sensitivity, and selectivity towards Cu^2+^ and Fe^3+^ ions, with LODs of 2.76 μM and 0.76 μM, respectively. These LODs were significantly lower than those reported in previous studies. Additionally, a comprehensive investigation was conducted to elucidate the potential photoluminescence mechanisms, which revealed that the interaction between the carboxyl group in the framework (acting as Lewis base site) and the metal ions Cu^2+^/ Fe^3+^ Lewis acid sites) may be responsible for competitive absorption (CA), resonance energy transfer (FRET), and photon electron transfer (PET) mechanisms. What's more, combining organic dyes with MOFs with stabilized structures is a very promising method for the recognition of Cu^2+^ and Fe^3+^, which paves the way for future investigation and practical applications in the detection of multicomponent metal ions.

### Supplementary Information

Supplementary Information.

## Data Availability

Data is provided within the manuscript or supplementary information files.
