# Supplementary material for: A dual-responsive RhB-doped MOF probe for simultaneous recognition of Cu2+ and Fe3+
Source: Sci Rep. 2024 May 23;14:11740. doi: 10.1038/s41598-024-62177-x (PMC11111689; doi:10.1038/s41598-024-62177-x)
Supplement: Supplementary file 1 — Supplementary Information. [file 41598_2024_62177_MOESM1_ESM.pdf]

Supplementary Information

A Dual-responsive RhB-doped MOF probe for Simultaneous Recognition of Cu^{2+} and Fe^{3+}

Teng Zhang,[1] Rui Cao,[1] Jingying Li,[1] Hanxiao Tang,[2] Hang Su,[1] Weisheng Feng,[1] Zhijuan Zhang, [*1, 3, 4]

1. College of Pharmacy, Henan University of Chinese Medicine, Zhengzhou 450046, China

2. College of Chinese Medical Sciences, Henan University of Chinese Medicine, Zhengzhou 450046, China

3. Collaborative Innovation Center of Research and Development on the Whole Industry Chain of Yu-Yao, Henan Province, Zhengzhou 450046, China

4. Institute of Mass Spectrometer and Atmospheric Environment, Jinan University, Guangzhou 510632, China

Corresponding author at Jinshui East Road 156, 450046, Zhengzhou, PR China.

E-mail: zhangyan0204@126.com

Materials and Methods

KCl, $ZrCl_4$, $CuCl_2·2H_2O$, $FeCl_3·6H_2O$, and Rhodamine B (RhB) were all bought from Aladdin. $AgNO_3$, $LiNO_3$, *N,N′*-dimethylformamide (DMF), biphenyl-4,4′-dicarboxylate (H_2BPDC), and ethanol (C_2H_5OH) were all purchased from J&K Scientific. $ZnCl_2$ and $BaCl_2$ were supplied by Macklin. $MnCl_2·4H_2O$, $Ni(NO_3)_2·6H_2O$, $CrCl_3·6H_2O$ and phenylarsine oxide (C_6H_5AsO) were obtained from RHAWN Chemicals. No further purification was performed on any chemicals obtained from commercial sources. The deionized water was obtained using the Milli-Q ultrapure water system. *Alisma plantago-aquatica* L. was purchased from a pharmaceutical company in Zhengzhou, Henan Province. Fourier transform infrared spectroscopy (FT-IR) was measured by Bruker TENSOR II recorded on Bruker D8 Advance Conventional Angle X-ray diffractometer. Scanning electron microscope (SEM) and energy dispersive X-ray spectroscopy were measured by TESCAN MIRA LMS. X-ray photoelectron spectra (XPS) were obtained by a Thermo Scientific ESCALAB 250Xi instrument (Thermo Scientific, America) with a monochromatic Al Kα X-ray source (1486.6 eV). Fluorescence lifetime was recorded on an Edinburgh FLS980 instrument by scanning at Ex=335 nm with a nanosecond lamp. The UV-ViS spectrum was measured by UNICO 4802s UV/ViS spectrophotometer. Fluorescence sensing was recorded by Hitachi F-7100 spectrophotometer.

Pore structure analysis

The BET surface area measurements of UiO-67 and RhB@UiO-67 were performed with N_2 adsorption/desorption isotherms at liquid nitrogen temperature (77 K) after degassing under vacuum at 423 K for 12 h using Quantachrome IQ_2 analyzer. The specific surface areas were calculated using the Brunauer-Emmett-Teller (BET) method in the p/p_0 range from 0.05 to 0.3. The pore size distribution curves were calculated using the adsorption branch of the isotherms and the nonlocal density functional theory (NLDFT) method. The micropore volume was calculated by using the t-plot method of Lippens and de Boer to the adsorption data, and the total pore volume was taken by a single-point method at p/p_0 = 0.99.

The calculation of energy transfer efficiency

The following equation calculates energy transfer efficiency:

$$E=1-\tau_{D\text{-}A}/\tau_D \tag{1}$$

Where τ_D is the fluorescence lifetime of the host before adding the guest molecule, and τ_{D-A} is the fluorescence lifetime of the host after adding the guest molecule.

Recyclability of RhB@UiO-67 (1:6)

In each cycle, the RhB@UiO-67 (1:6) material was subjected to a series of purification steps, including centrifugation, ethanol soaking, and sonication, following the detection of Cu^{2+} or Fe^{3+}. These steps were repeated until the absence of visible Cu^{2+} or Fe^{3+} coloration. Subsequently, the dried RhB@UiO-67 (1:6) material was assessed for reusability and sensing capabilities in the next cycle.

Figure captions

Fig. S1(a) SEM image of UiO-67

Fig. S1(b) EDS mapping of UiO-67

Fig. S1(c) SEM image of RhB@UiO-6 (1:6).

Fig. S1(d) EDS mapping of RhB@UiO-6 (1:6).

Fig. S2 PXRD patterns of RhB@UiO-67 materials.

Fig. S3 TGA curves of UiO-67 and RhB@UiO-67 series

Fig. S4(a) The solid-state fluorescence spectra of H_2BPDC (λex=333 nm), UiO-67 (λex=333 nm), RhB@UiO-67 (1:6) (λex=333 nm)

Fig. S4(b) The solid-state fluorescence spectra of RhB@UiO-67 in different proportions

Fig. S5(a) Fluorescence emission spectra of RhB@UiO-67(1:6) in different solvents (λex=335 nm)

Fig. S5(b) Different concentrations of RhB@UiO-67(1:6) Fluorescence spectra in 90% methanol solution (λex=335 nm)

Fig. S5(c) Fluorescence intensity of RhB at different RhB@UiO-67(1:6).

Fig. S5(d) Fluorescence intensity of BPDC at different RhB@UiO-67(1:6).

Fig. S6(a) Relative fluorescence intensity of RhB@UiO-67(1:6) at different times (0.33 mg/mL)

Fig. S6(b) The relative fluorescence intensity of RhB@UiO-67(1:6) in the pH range of 1-10

Fig. S6(c) The relative fluorescence intensity of RhB@UiO-67(1:6) soaked in ethanol for one week at room temperature.

Fig. S7(a) Fluorescence emission spectra of RhB@UiO-67(1:6) (λex=335 nm, 0.33 mg/mL) sensing (10^{-3} moL/L) Cu^{2+}, Fe^{3+}, and Cu^{2+} with Fe^{3+}.

Fig. S7(b) Fluorescence response of UiO-67 (λex=322 nm) (0.33 mg/mL) in the presence of various metal ions.

Fig. S8(a) Fluorescence spectra of UiO-67 (λex = 322 nm) in the presence of various concentrations of Cu^{2+} in ethanol solution (the inset shows the CIE chromaticity diagram)

Fig. S8(b) linear plot of UiO-67 (λex = 322 nm) in the presence of various concentrations of Cu^{2+} in ethanol solution (the inset shows the CIE chromaticity diagram)

Fig. S8(c) Fluorescence spectra of UiO-67 (λex = 322 nm) in the presence of various concentrations of Fe^{3+} in ethanol solution (the inset shows the CIE chromaticity diagram).

Fig. S8(d) linear plot of UiO-67 (λex = 322 nm) in the presence of various concentrations of Fe^{3+} in ethanol solution (the inset shows the CIE chromaticity diagram).

Fig. S9(a) N_2 adsorption/desorption isotherms at 77 K of RhB@UiO-67 (1:6) after 5 cycles of regeneration.

Fig. S9(b) NLDFT pore size distributions of RhB@UiO-67 (1:6) after 5 cycles of regeneration.

Fig. S9(c) N_2 adsorption/desorption isotherms at 77 K of RhB@UiO-67 (1:6) after 8 cycles of regeneration.

Fig. S9(d) NLDFT pore size distributions of RhB@UiO-67 (1:6) after 8 cycles of regeneration.

Fig. S10(a) The infrared spectrum of RhB@UiO-67 (1:6) in the range of 400-4000 cm^{-1} after being immersed in Cu^{2+} and Fe^{3+} ethanol solution for 24 h.

Fig. S10(b) The infrared spectrum of RhB@UiO-67 (1:6) in the range of 400-1800 cm^{-1} after being immersed in Cu^{2+} and Fe^{3+}ethanol solution for 24 h.

Fig. S11(a) The high-resolution XPS spectra of samples: Cu 2p

Fig. S11(b) The high-resolution XPS spectra of samples: Fe 2p

Fig. S11(c) The high-resolution XPS spectra of samples: O 1s

Fig. S12(a) The fluorescence lifetime (λem = 392 nm) of RhB@UiO-67 (1:6) after adding different concentrations of Cu^{2+}

Fig. S12(b) The fluorescence lifetime (λem = 581 nm) of RhB@UiO-67 (1:6) after adding different concentrations of Cu^{2+}

Fig. S12(c) Fluorescence lifetime (λem = 392 nm) of RhB@UiO-67 (1:6) after adding different concentrations of Fe^{3+}

Fig. S12(d) Fluorescence lifetime (λem = 581 nm) of RhB@UiO-67 (1:6) after adding different concentrations of Fe^{3+}

Table captions

Table S1 Comparison of some reported approaches for Cu(II) and Fe(III) detection

Table S2 Pore structure parameters of the ratio fluorescence probe RhB@UiO-67(1:6) after multiple cycles of regeneration

Table S3 Fluorescence lifetime parameters after adding Cu^{2+} at different emission wavelengths

Table S4 Fluorescence lifetime parameters after adding Fe^{3+} at different emission wavelengths

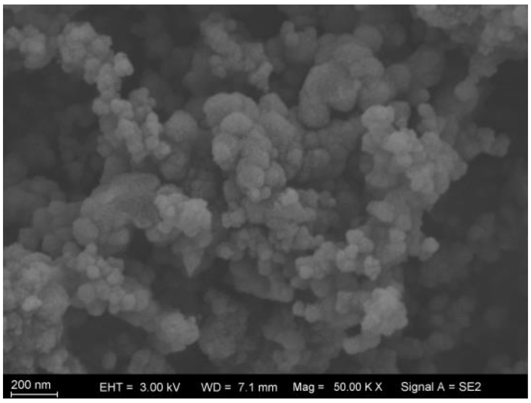

(a)

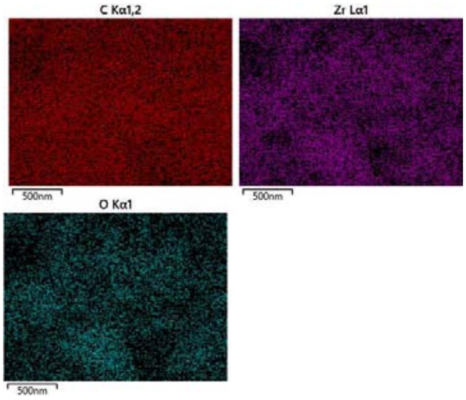

(b)

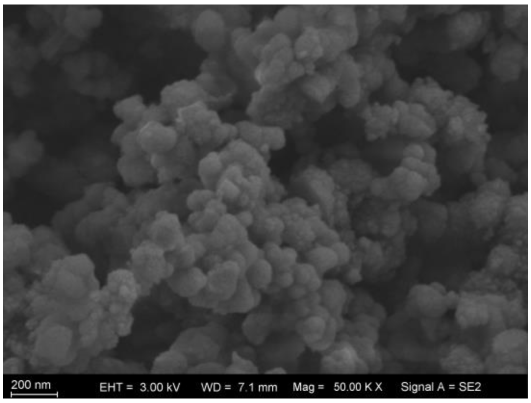

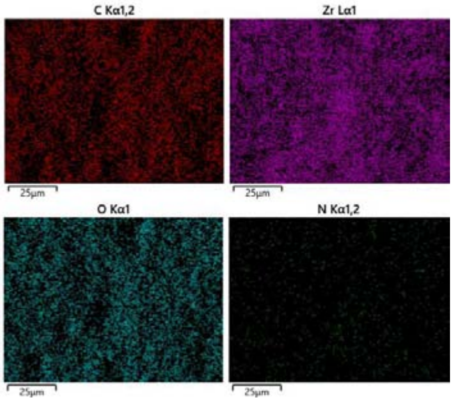

(c) **(d)**

Fig. S1 (a) SEM image and **(b)** EDS mapping of UiO-67. **(c)** SEM image and **(d)** EDS mapping of RhB@UiO-6 (1:6).

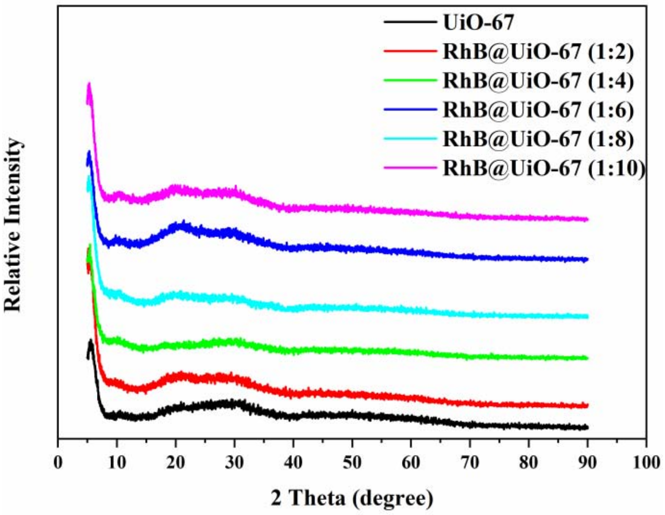

Fig. S2 PXRD patterns of RhB@UiO-67 materials.

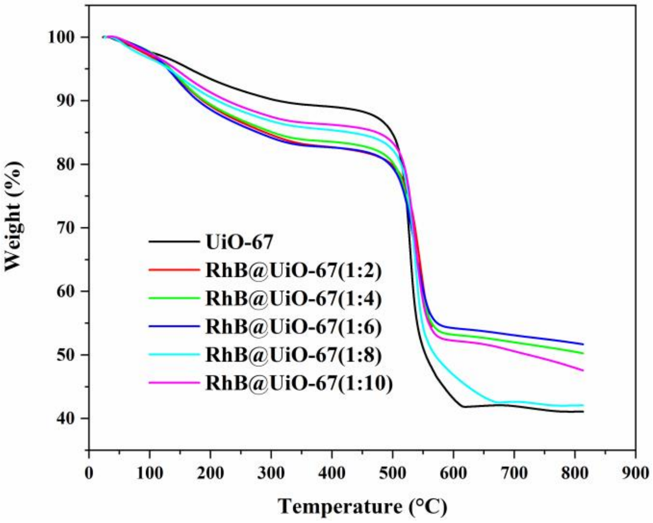

Fig. S3 TGA curves of UiO-67 and RhB@UiO-67 series

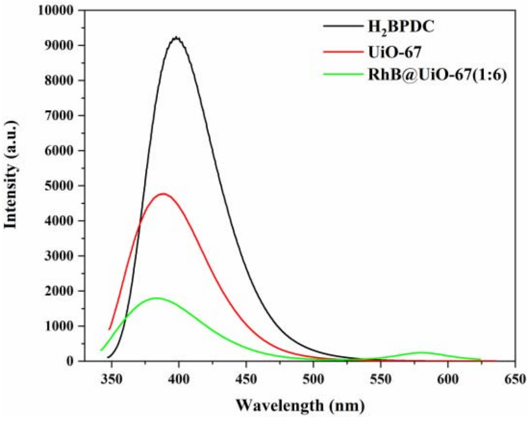

(a)

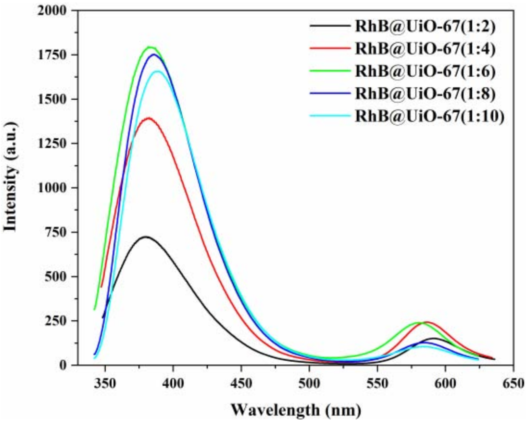

(b)

Fig. S4 The solid-state fluorescence spectra of **(a)** H_2BPDC (λex=327 nm), UiO-67 (λex=328 nm), RhB@UiO-67 (1:6) (λex=322 nm); **(b)** RhB@UiO-67 in different proportions

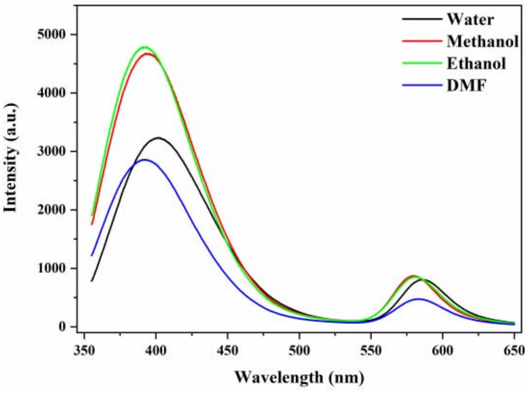

(a)

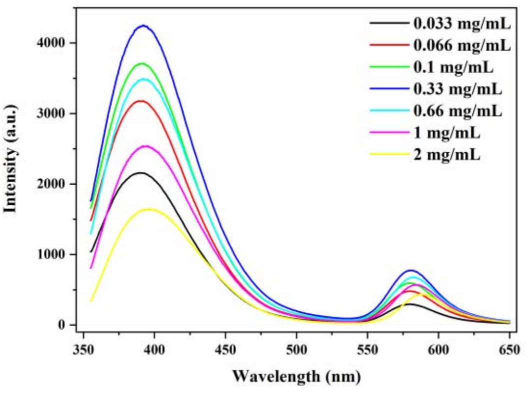

(b)

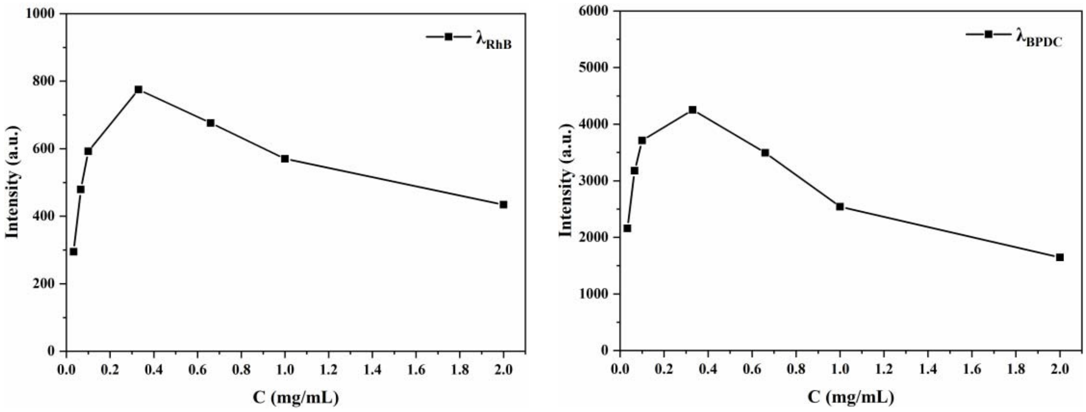

(c) **(d)**

Fig. S5 (a) Fluorescence emission spectra of RhB@UiO-67(1:6) in different solvents (λex=335 nm); **(b)** Different concentrations of RhB@UiO-67(1:6) Fluorescence spectra in 90% methanol solution (λex=335 nm); Fluorescence intensity of **(c)** Rh and **(d)** BPDC at different RhB@UiO-67(1:6).

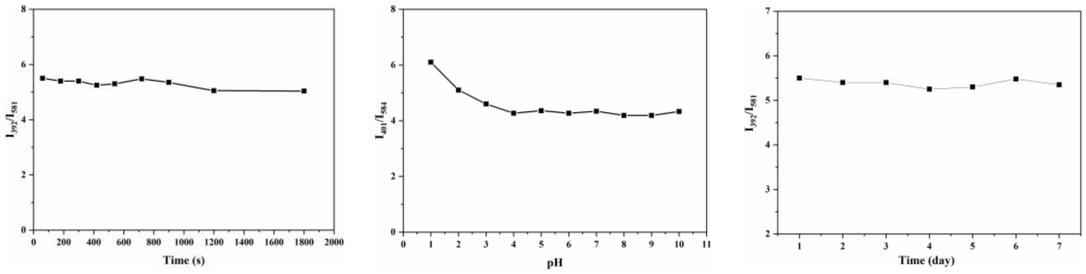

(a) **(b)** **(c)**

Fig. S6 (a) Relative fluorescence intensity of RhB@UiO-67(1:6) at different times (0.33 mg/mL); **(b)** The relative fluorescence intensity of RhB@UiO-67(1:6) in the pH range of 1-10; **(c)** The relative fluorescence intensity of RhB@UiO-67(1:6) soaked in ethanol for one week at room temperature.

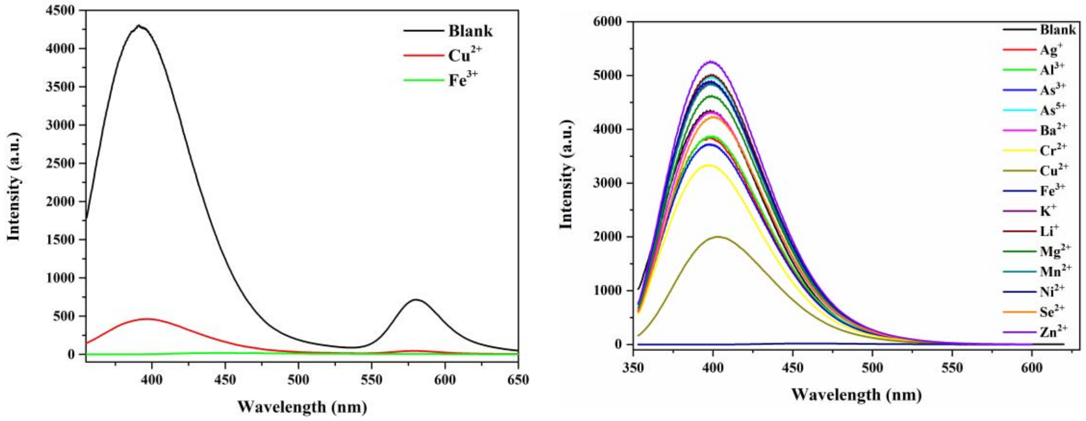

(a) **(b)**

Fig.S7 **(a)** Fluorescence emission spectra of RhB@UiO-67(1:6) (λex=335 nm, 0.33 mg/mL) sensing (10^{-3} moL/L) Cu^{2+}, Fe^{3+}, and Cu^{2+} with Fe^{3+}. **(b)** Fluorescence response of UiO-67 (λex=330 nm) (0.33 mg/mL) in the presence of various metal ions.

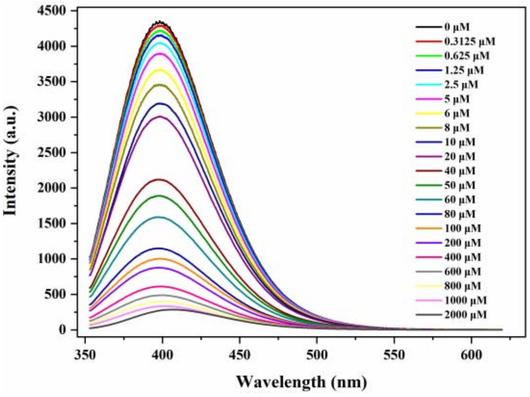

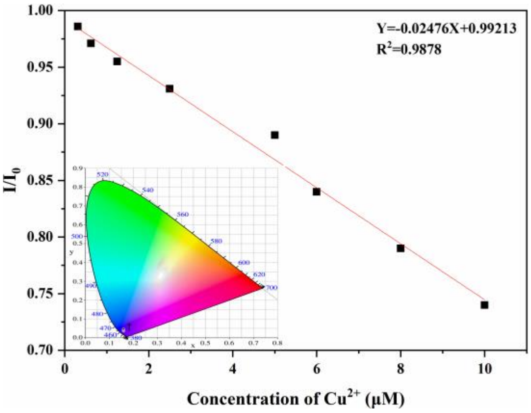

(a) **(b)**

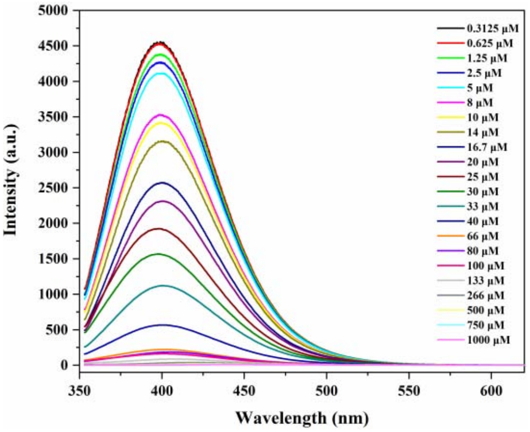

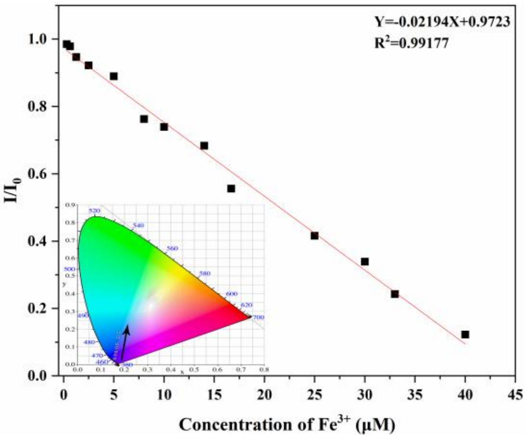

(c) **(d)**

Fig. S8 **(a)** Fluorescence spectra and **(b)** linear plot of UiO-67 (λex = 322 nm) in the presence of various concentrations of Cu^{2+} in ethanol solution (the inset shows the CIE chromaticity diagram); **(c)** Fluorescence spectra and **(d)** linear plot of UiO-67 (λex = 330nm) in the presence of various concentrations of Fe^{3+} in ethanol solution (the inset shows the CIE chromaticity diagram).

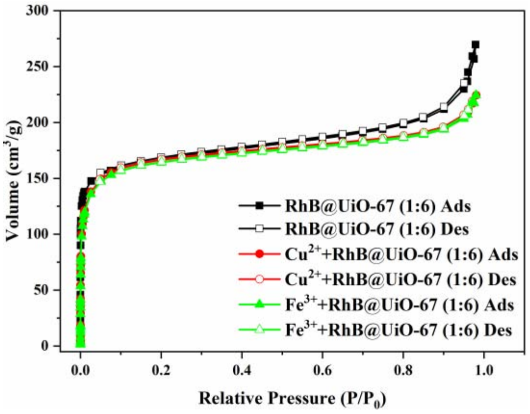

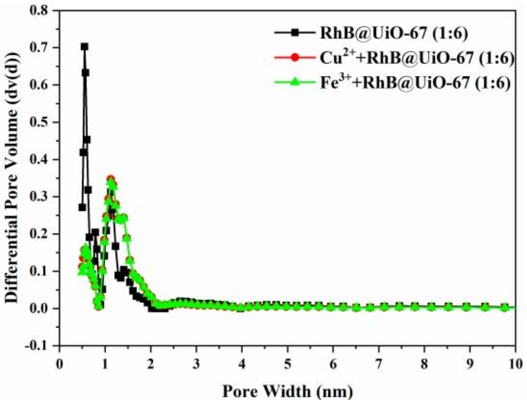

(a) **(b)**

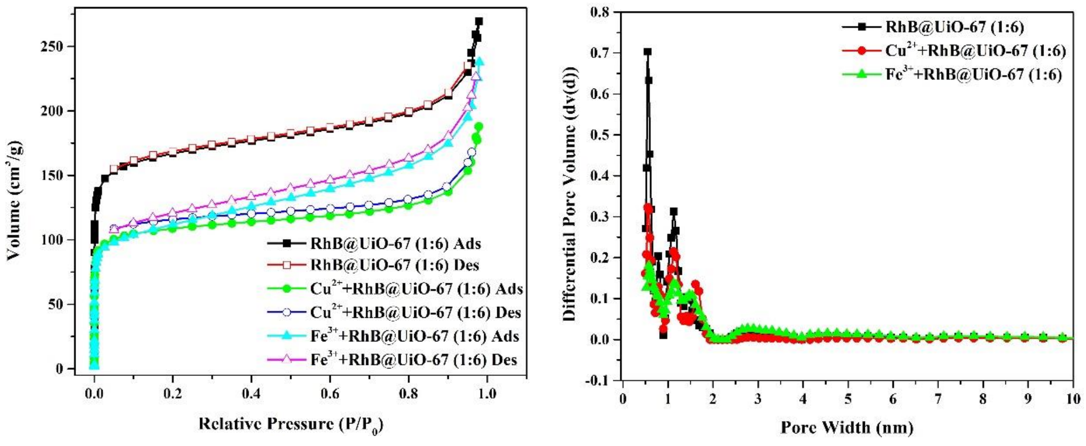

(c) (d)

Fig. S9 (a) N_2 adsorption/desorption isotherms at 77 K and **(b)** NLDFT pore size distributions of RhB@UiO-67 (1:6) after 5 cycles of regeneration; **(c)** N_2 adsorption/desorption isotherms at 77 K and **(d)** NLDFT pore size distributions of RhB@UiO-67 (1:6) after 8 cycles of regeneration;

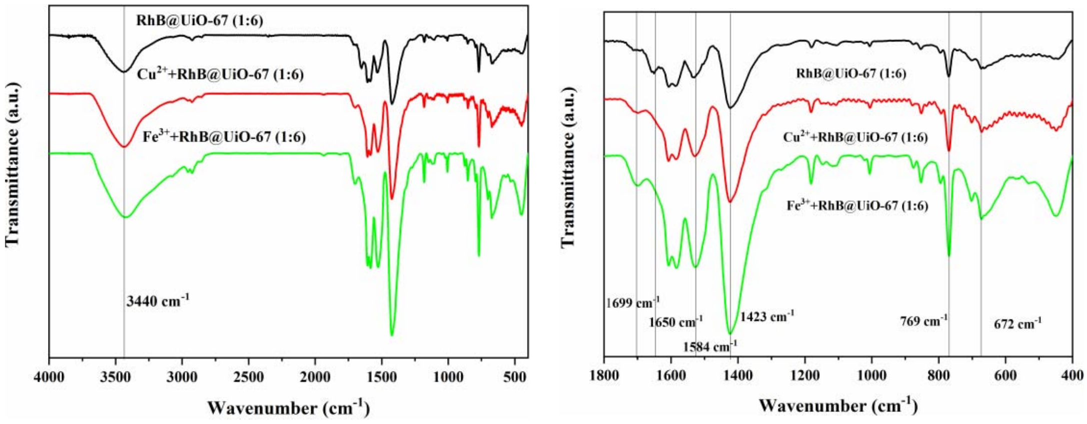

(a) (b)

Fig. S10 (a) The infrared spectrum of RhB@UiO-67 (1:6) in the range of 400-4000 cm^{-1} after being immersed in Cu^{2+} and Fe^{3+} ethanol solution for 24 h. **(b)** The infrared spectrum of RhB@UiO-67 (1:6) in the range of 400-1800 cm^{-1} after being immersed in Cu^{2+} and Fe^{3+}ethanol solution for 24 h.

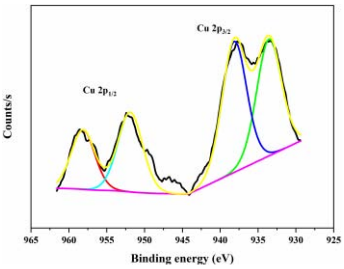

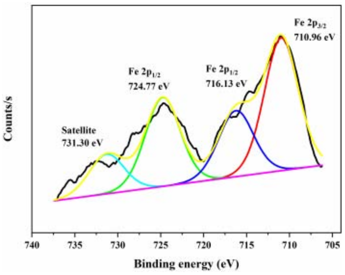

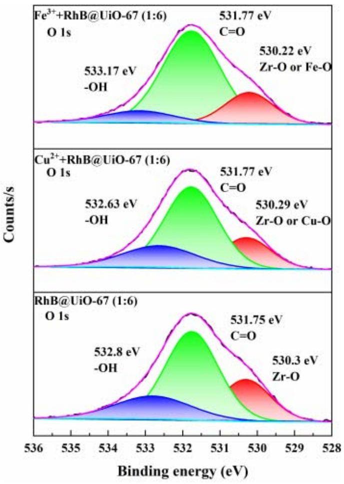

(a) **(b)** **(c)**

Fig. S11 The high-resolution XPS spectra of samples: **(a)** Cu 2p, **(b)** Fe 2p, and **(c)** O 1s

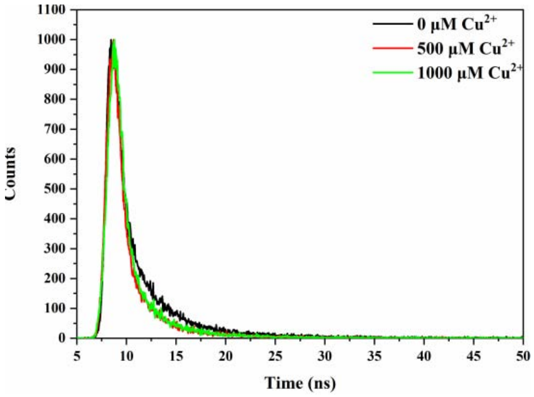

(a)

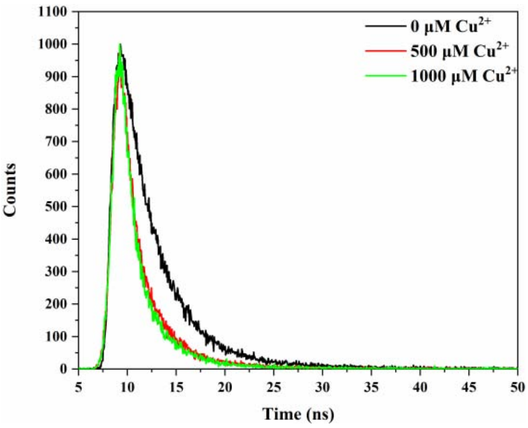

(b)

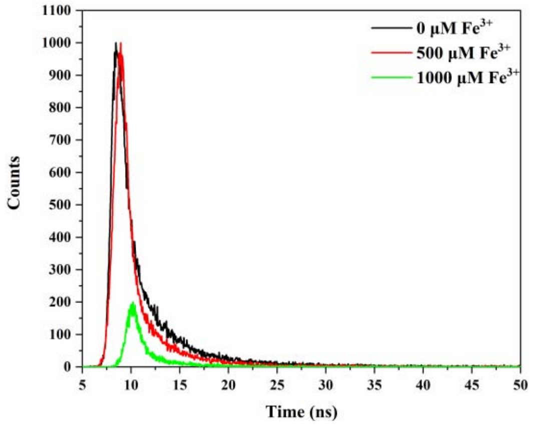

(c)

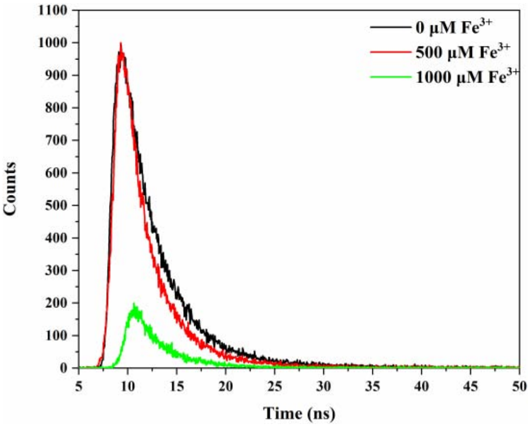

(d)

Fig. S12 The fluorescence lifetime **(a)** (λem = 392 nm) and **(b)** (λem = 581 nm) of RhB@UiO-67 (1:6)after adding different concentrations of Cu^{2+}; Fluorescence lifetime **(c)** (λem = 392 nm) and **(d)** (λem = 581 nm) of RhB@UiO-67 (1:6) after adding different concentrations of Fe^{3+};

Table S1 Comparison of some reported approaches for Cu(II) and Fe(III) detection

NO.	Materials	method	Linear range (μM)	LOD (μM)	Ref.
1	Ag/Zn-ZIF-8	PL	1-10	3.9 (Fe^{3+})<br>6.7(Cu^{2+})	1
2	{[Cd_2(dpc)(bib)(H_2O)]·H_2O}n	PL	0-100	8.9 (Fe^{3+})<br>49.9 (Cu^{2+})	2
3	Eu^{3+}@CAU-11	PL	1-10	7.8 (Fe^{3+})<br>6.2 (Cu^{2+})	3
4	Tb^{3+}@Ni-BTC	PL	1–40	2.82 (Fe^{3+})	4
5	AgNPs	colorimetry	100-500	195.24 (Fe^{3+})	5
6	o-HBA AuNPs	Colorimetry	0–1000	9.19 (Fe^{3+})	6
7	$MoSe_2$@Fe enzyme	PL/COL	PL 25–300<br>COL 5–80	PL 1.97<br>COL 0.65<br>(Fe^{3+})	7
8	T-CDs	PL/COL	PL 0–50<br>COL 0–50	PL 2.78<br>COL 3.39<br>(Fe^{3+})	8
9	Cd/Zr-UiO-66 (1:9)	PL	0-10	4.3(Fe^{3+})	9
10	Eu-MOF	PL	0-16	13.2<br>(Fe^{3+})	10
11	RhB@UiO-67(1:6)	PL	0-35	25.3 (Cu^{2+})	This work
		PL	0-5	0.76<br>(Fe^{3+})	

Table S2 Pore structure parameters of the ratio fluorescence probe RhB@UiO-67(1:6) after multiple cycles of regeneration

Number of cycles	MOFs	BET (m^2/g)	Langmuir (m^2/g)	Micropore volume (cm^3/g)	Total pore volume (cm^3/g)	Average pore diameter (nm)
5	RhB@UiO-67(1:6)	641	754	0.219	0.417	2.599
	RhB@UiO-67(1:6) + Cu^{2+}	640	749	0.226	0.347	2.168
	RhB@UiO-67(1:6) + Fe^{3+}	631	744	0.225	0.347	2.197
8	RhB@UiO-67(1:6) + Cu^{2+}	420	512	0.149	0.291	2.765
	RhB@UiO-67(1:6) + Fe^{3+}	412	494	0.113	0.367	3.570

Table S3 Fluorescence lifetime parameters after adding Cu^{2+} at different emission wavelengths

λEm	Concentration (μM)	Fluorescence Lifetime (τ)	Energy transfer efficiency	χ2
	0	3.20	--	1.0626
392	500	2.32	27.5%	0.9405
	1000	2.30	28.1%	0.9371
	0	3.90	--	1.0626
581	500	2.76	29.2%	0.9486
	1000	2.52	35.4%	0.9604

Table S4 Fluorescence lifetime parameters after adding Fe^{3+} at different emission wavelengths

λEm	Concentration (μM)	Fluorescence Lifetime (τ)	Energy transfer efficiency	χ2
	0	3.20	--	1.0626
392	500	2.39	25.1%	0.9335
	1000	1.57	50.9%	1.1024
	0	3.90	--	1.0626
581	500	3.30	15.4%	1.1381
	1000	2.98	23.6%	0.8864

Notes and references

1. Geng R, Tang H, Ma Q, Liu L, Feng W, Zhang Z. Bimetallic Ag/Zn-ZIF-8: An efficient and sensitive probe for Fe^{3+} and Cu^{2+} detection. Colloids Surf A Physicochem Eng Asp 632: 127755.https://doi.org/10.1016/j.colsurfa.2021.127755 (2022).
2. Du Y, Yang H, Shao C, Liu J, Yan Y, Yu L, Zhu D, Huang C, Yang L. Three d^{10} based metal-organic frameworks constructed from 2-(3',4'-dicarboxylphenoxy) isophthalic acid: Dual-functional luminescent sensors for Cu^{2+}, Fe^{3+} cations and Aspartic acid. J Solid State Chem 277:564-574.https://doi.org/10.1016/j.jssc.2019.07.012 (2019)
3. Zhang Y, Yan B. A ratiometric fluorescent sensor with dual response of Fe^{3+}/Cu^{2+} based on europium post-modified sulfone-metal-organic frameworks and its logical application. Talanta 197:291-298.https://doi.org/10.1016/j.talanta.2019.01.037 (2019).
4. Chen X, Qi C, Li H, Ding J, Yan S, Lei H, Xu L, Liu B. Highly sensitive and selective Fe^{3+} detection by a water-stable Tb^{3+}-doped nickel coordination polymer-based turn-off fluorescence sensor. J Solid State Chem 281: 121030.https://doi.org/10.1016/j.jssc.2019.121030 (2020).
5. Moond M, Singh S, Sangwan S, Devi P, Beniwal A, Rani J, Kumari A, Rani S. Biosynthesis of

silver nanoparticles utilizing leaf extract of trigonella foenum-graecum l. For catalytic dyes degradation and colorimetric sensing of Fe^{3+}/Hg^{2+}. Molecules 28:951.https://doi.org/10.3390/molecules28030951 (2023).

6. Andreani A, Kunarti E, Hashimoto T, Hayashita T, Santosa S. Fast and selective colorimetric detection of Fe^{3+} based on gold nanoparticles capped with ortho-hydroxybenzoic acid. J Environ Chem Eng 9: 105962.https://doi.org/10.1016/j.jece.2021.105962 (2021).
7. Lin L, Chen D, Lu C, Wang X. Fluorescence and colorimetric dual-signal determination of Fe^{3+} and glutathione with $MoSe_2$@Fe nanozyme. Microchem J 177: 107283.https://doi.org/10.1016/j.microc.2022.107283 (2022).
8. Jiang T, Huang J, Ran G, Song Q, Wang C. A colorimetric and fluorometric dual-mode carbon dots probe derived from phenanthroline precursor for the selective detection of Fe^{2+} and Fe^{3+}. Anal Sci 39:325-333.https://doi.org/10.1007/s44211-022-00236-x (2023).
9. Geng R, Li P, Tang H, Liu L, Huang H, Feng W, Zhang Z. Bimetallic Cd/Zr-UiO-66 material as a turn-on/off probe for As^{5+}/Fe^{3+} in organic media. Chemosphere 291: 132827.https://doi.org/10.1016/j.chemosphere.2021.132827 (2022)
10. Du Y, Yang H, Liu R, Shao C, Yang L. A multi-responsive chemosensor for highly sensitive and selective detection of Fe^{3+}, Cu^{2+}, $Cr_2O_7^{2-}$ and nitrobenzene based on a luminescent lanthanide metal–organic framework. Dalton Trans 49:13003-13016.https://doi.org/10.1039/d0dt02120b (2020).
